# Increasing yield stability and input efficiencies with cost-effective mechanization in Nepal

**DOI:** 10.1016/j.fcr.2018.08.012

**Published:** 2018-11-01

**Authors:** Alex G. Park, Andrew J. McDonald, Mina Devkota, Adam S. Davis

**Affiliations:** a Department of Crop Sciences, University of Illinois at Urbana-Champaign, S-306 Turner Hall, 1102 South Goodwin Avenue, Urbana, IL 61801, USA; b CIMMYT-Nepal, Singh Durbar Plaza Marg, Kathmandu, Nepal

**Keywords:** Nepal, Wheat (*T. aestivum*), Mechanization, Chest-mounted spreader, Hand distributed inputs

## Abstract

Nepal is at a crossroads of diminishing farm-labor and inadequate investment into farming operations that, among other factors, have stagnated domestic wheat yield. Cultural and economic constraints have hindered the widespread adoption of more expensive precision agriculture technologies like zero-till that have the capacity to improve labor and farm input efficiencies. To capture the benefits from added precision of application but with the ability to fit within the current semi-mechanized seed bed preparation and tillage system, we introduced a low-cost, chest mounted seed and fertilizer. We found that simple mechanization caused yield efficiencies to be positive and significant for nitrogen and phosphate. Seed rates using this method were positively associated with seedling density. This led to both yield and profit being more predictable for farmers. Conversely, hand-applied inputs caused a disassociation between inputs and end of season yield and therefore added a large measure of risk to their farming operations.

## Highlights

• Mechanization provided more predictable profit and return on investment of inputs.
• Applying inputs by hand disassociated fertilizer from end of season yield.
• Chest-mounted spreader can be stop-gap between hand distributed inputs and zero-till.
• Labor efficiency was roughly doubled by using chest-mounted spreader.
• Chest-mounted spreader increased within and between-field uniformity of yield.

## 1 Introduction

Nepal has the lowest cereal yield per hectare among the south Asian countries that provide the region with its domestic source of grain. The cause of Nepal’s low yields has been attributed to a stunting of agricultural intensification caused by short-sighted development policies and socioeconomic crises (Karan et al., 1994; Sharma, 2006). Limited adoption of agronomic practices by farmers that increase yield are on a collision course with a diminishing labor market that will further undermine domestic food security if not addressed (Joshi et al., 2012; Seddon et al., 2002). Long-term solutions to these problems will require policy changes at the national level by the Nepali government, while more immediate solutions can be found by targeting appropriate technologies at ineffective agricultural practices. Here we document the effects of low-cost, simple mechanization (in the form of a chest-mounted seed and fertilizer spreader) on yield, yield variability, efficiencies and others metrics compared to the traditional hand application of inputs.

Stagnation of agricultural intensification in Nepal has exposed farmers to risk by preventing them from adopting better agronomic practices like appropriate management of soil fertility. Fertilizer rates for nitrogen (N), phosphate (P), and potassium (K) on the Terai of Nepal –the most productive and developed agricultural region adjacent India– are 40%, 26%, and 70% less, respectively compared to farmers in neighboring Bihar, India (Park et al., 2018). When fertilizer is applied, 75–80% of it comes from gray market sources from India (Pandey, 2014). The effects of inadequate supplies of affordable fertilizer to crop productivity in Nepal are compounded by decreasing availability of agricultural labor (Maharjan et al., 2013).

In response to limited opportunities for economic advancement in farming, agricultural laborers and farmers in the 1990s began leaving the sector en masse in search of more lucrative work abroad (Central Bureau of Statistics, 2009; Seddon et al., 2002). This trend has only accelerated, with 10% of the Nepali population working overseas in the remittance economy by 2014 (Kaphle, 2014; NIDS, 2018). This departure of farm labor was found to dramatically reduce the productivity of Nepali agriculture on a farm by farm basis. For every laborer that left a household in which they were part of the labor pool, total crop productivity dropped 11% (Maharjan et al., 2013). As labor becomes scarcer in the Nepali agricultural economy, labor bottlenecks have emerged as an increasing problem. Labor bottlenecks occur when there are labor shortages, and are especially problematic during critical times of agricultural operations (Pingali, 2007). Bottlenecks often occur around seed bed preparation, sowing, top dressing and harvesting. Delays in these operations have significant consequences to the productivity of the wheat system in Nepal and South Asia. A common example of a labor bottleneck in Nepal is the late sowing of wheat. Delays in sowing can reduce yields by 0.7% for every day delayed past an optimum sowing window due to late season heat stress (Ortiz-Monasterio et al., 1994). Solutions to labor bottlenecks increasingly take the form of mechanization, or technology more broadly, in most global agricultural systems (Pingali, 2010).

Immediate solutions to the specific problems of labor and fertilizer scarcity can be undertaken using technology that increase efficiencies. A technological solution that focuses on improving efficiency of inputs and labor best reflects the reality that an increase of both inputs and labor in Nepal is unlikely to increase in the near future because of the long-term political and socioeconomic roots of these problems (Sharma, 2006). To have a realistic chance of adoption at scale, technological solutions must be low-cost, simple for easy maintenance, and capable of fitting within the status quo of agricultural practices of Nepal. These criteria are part of successful agricultural development projects in the past that adapted appropriate technologies to the constraints of the local agricultural systems (ATTRA, 2018). Past development projects in Nepal that leveraged advanced agricultural technologies have often failed in the long-term because the supporting manufacturing, machinery, and agribusiness sectors were unable to maintain complex equipment or processes after the initial support for the introduction of the technology was completed (Maharjan et al., 2013; Metz, 1995).

A source of inefficiency in Nepal ripe for improvement with an appropriate technological intervention is the traditional practice of applying farm inputs by hand. We believe this traditional practice is a principal source of within-field variability and, we hypothesize, a prime contributor to resource use inefficiencies and yield gaps. An intervention that increases the precision and speed of application of seed and fertilizer would improve both input and labor efficiencies. We therefore sought to test if a simple, chest-mounted spreader could improve the following aspects of the farming system in our study relative to traditional methods: 1) improve uniformity of wheat yield within fields, 2) improve fertilizer efficiency of nitrogen and phosphate with respect to yield and an independent measure of crop vigor, 3) increase seed efficiency to seedling establishment and yield, and 4) increase labor efficiency. We then assessed whether the net effect of mechanization provided meaningful improvements to a farmer’s return on investments.

## 2 Methods

### 2.1 Overview

To test whether simple mechanization could improve fertilizer and seed efficiency compared to traditional hand applied methods on the Terai of Nepal, we split a group of 60 farmer participants into two treatment groups within a Completely Randomized Design. Thirty farmers received an application of farm inputs using a chest-mounted spreader, while the other 30 applied these inputs by hand.

### 2.2 Study location and timing

The study area was located near the town of Siddharthanagar in the district of Rupandehi in the Terai region of Nepal (27.5126 °N, 83.4816 °E) where the dominant annual cropping pattern is a rice-wheat rotation (Mahajan and Gupta, 2009). Trials began in November of 2016 with sowing and concluded in April 2017 when harvested. The study area climate is sub-tropical, with a mean annual temperature between 20 and 25 °C and an average annual rainfall of approximately 1400 to 2000 mm (WFP, 2010) which mostly falls during monsoon. All fields in the study received at least one irrigation during the wheat growing season.

### 2.3 Technological intervention and traditional practices

We selected a chest-mounted spreader as our intervention to apply the granular inputs of urea, diammonium phosphate, and seed to farmer’s fields. The model chosen was a (*Model 2750, Manufacturer*-*EarthWay)* spreader, commonly used to fertilize lawns in America and Europe. An agitator feeds granular material from a top mounted nylon hopper to the distribution plate where it is spread in a fanning action of approximately 45° in front of the user’s chest who controls rate of application through speed of cranking and a flow control mechanism. Inputs were applied by travelling along the perimeter of the field with the left side of the fan overlapping the right side of the previous pass (Wolf and Smith, 1979). The current price for a single unit sold in the United States at the time of publication was $35 USD. This simple device was compared to the traditional method of applying fertilizer and seed by hand. In the traditional method, fertilizer or seed is placed in a container, which is applied by hand as the laborer walks up and down a field applying the input as uniformly as possible. Under both mechanized and traditional treatments, the inputs were then incorporated by either a cultivator or rotovator.

### 2.4 Experimental design and input rates

Sixty farmers were selected at random for inclusion in a Completely Randomized Design trial, with the two treatments applied to 30 farms each. A single researcher applied farm inputs with the spreader, while farmers applied inputs to their own fields. Within each farmer’s field, four 1 m^2^ subsamples were randomly established to capture heterogeneity of response variables across the season. As these were on-farm trials, researchers only controlled different application techniques of seed and fertilizer. All farmers were provided 3.75 kg of diammonium phosphate, and 4 kg of urea after it was determined that many farmers in the trials would have no fertilizer to apply whatsoever because of inadequate access or funding, thereby making the experiment irrelevant. If farmers were able to afford fertilizer, they almost always added the amount we provided them to their own supply, thereby increasing their rates (information that we recorded). The rates of fertilizer in these trials for N and P are 21% higher than those in a recent production survey (Park et al., 2018), and reflect the combining of farmer fertilizer with that provided by researchers. Seed was provided by farmers and represented 12 unique varieties. Field sizes ranged between 0.014 ha to 0.11 ha, and averaged 0.04 ha.

### 2.5 Normalized difference vegetation index, end of season yield estimates, and seedling density

Normalized Difference Vegetation Index (NDVI) was recorded bi-weekly throughout the wheat season at all subsamples because of its strong relationship with both plant uptake of fertilizer (Teal et al., 2006) and end of season yield (Wiegand and Richardson, 1990). Time constraints at the time of harvest necessitated a four-step model approach to estimate yield at all subsamples in farmers’ fields. First, we harvested a single random subsample from each of the 60 fields within the study for an estimation of real yield. The yield of this sample was corrected for moisture content using a *wile 55* moisture meter. Second, we fit a quadratic model to the seasonal NDVI curves with random effects in the intercept and linear term for each farm, and a random effect in the intercept for each subsample. Third, we estimated the seasonal maximum NDVI using these fitted curves for each subsample because of its strong relationship to end of season yield (Labus et al., 2002). Fourth, simple linear models were fit between maximum NDVI values and the real yield values from the harvested subsample stratified by variety to allow for adequate replication. The resulting predictions of final yield were used as the response variable in this study. Seedling density was determined by visual counts within each of the subsamples. Variability of seedling density was determined by calculating the variance of all four subsamples within a given field.

### 2.6 Seasonally integrated normalized difference vegetation index

We calculated the area under the curve of an NDVI time series throughout the season to estimate seasonally integrated NDVI per sub-sample (R Core Team, 2017). Seasonally integrated NDVI is measure of crop vigor that is a strong proxy between fertilizer uptake and end of season biomass production and yield (Labus et al., 2002; Teal et al., 2006). We used values of seasonally integrated NDVI as a reasonable intermediary between crop vigor related to fertilizer uptake, fertilizer rates and end of season yield to better understand the mechanisms by which a more precise application of inputs affected wheat productivity.

### 2.7 Farmer partial-profit

The costs of inputs within the study were $0.18 kg^−1^ urea, $0.32 kg^−1^ diammonium phosphate, and $0.28 kg^−1^ seed. We assumed the values of seed to be that of the wholesale price of wheat grain at the end of the 2016–2017 wheat season. Our calculation of profit was determined by multiplying the yield per hectare of a farmer by the wholesale price of wheat grain minus inputs of N, P and seed. Our calculation of profit is therefore only a partial measure of profit, because we were unable to measure the costs of other inputs such as irrigation, machinery, diesel, etc.

### 2.8 Bardiya time trials

A separate Completely Randomized Design trial was established to determine whether there was an improvement in labor efficiency between mechanized versus hand distributed treatments in Bardiya district on the Terai of Nepal in November 2015. Forty farmers were split between the two treatments, with each receiving the equivalent rate of 120 kg ha^−1^ in seed to be applied to fields. Each treatment was timed from beginning to completion of application of seed.

### 2.9 Environmental data

Soil and atmospheric data were collected in an effort to control for their potential influence on interpretation of any interaction effects from the treatments. Bi-weekly volumetric soil moisture to a 1-meter depth was recorded through the growing season across all 60 farms, allowing for an estimation of the total seasonal abstraction of water through the soil profile. To account for potential differences in the effect of water stress, the Crop Water Stress Index was estimated at each subsample as soon as the wheat canopy closed till the initiation of senescence (Donald *J*. Garrot et al., 1994). A simple linear model was fit per field using the average of the Crop Water Stress Index values across the four sub-samples as the response variable plotted through time. The slope of the Crop Water Stress Index through time for each field gave an indication of the influence that water stress may have had on final yield, with greater slopes indicating a larger water stress and vice versa. A single weather station was installed within 1 km of the field sites to measure rainfall and heat throughout the growing season.

### 2.10 Statistical analysis

Linear mixed effects models were used to determine slope by interaction effects for the two treatments (Pinheiro et al., 2017). These models were used to test for treatment effects between N, P and yield and seasonally integrated NDVI, seed rate and yield and density, N, P, and seed rates to partial-profit. We used a single random effect for the intercept of sub-sample within farm. General linear models were used to find slope by interaction effects by treatment for seed rate on the variability of seedling germination, and input costs on profit (R Core Team, 2017). A simple linear model was fit between the variability of seasonally integrated NDVI and yield. Partial correlations were used to calculate the pairwise partial correlation between two variables while controlling for the third variable (Kim, 2015). Variances and averages were determined for each field using the four sub-samples therein for yield and seasonally integrated NDVI. The coefficient of variation was used to determine if there were significant differences between treatments among the variability of yield, seasonally integrated NDIV, seedling density, and partial-profit (Krishnamoorthy and Lee, 2014). Analysis of Variance was used to test whether the time of application between two treatments were significantly different from each other and differences between treatment groups in amount of inputs.

## 3 Results

### 3.1 Stand uniformity and yield stability

**Fig. 1:**
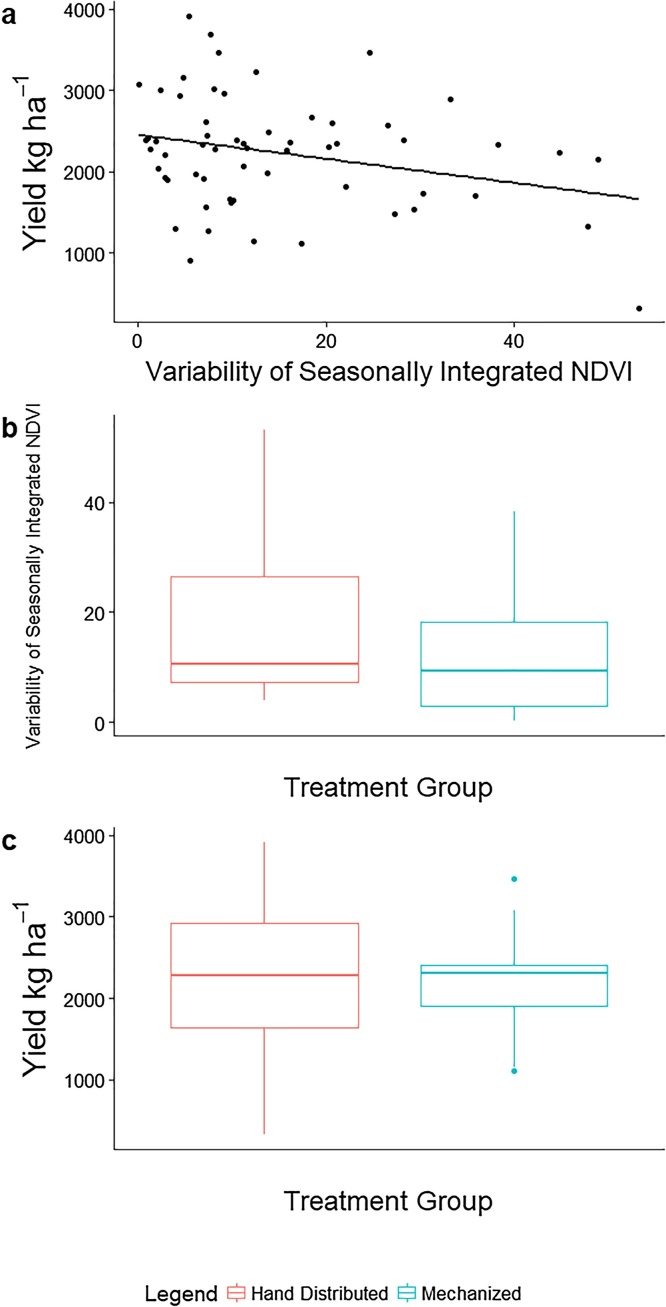
a) An increase in variability of seasonally integrated NDVI within a farmer’s field is associated with a decrease in yield, 2) The variability of seasonally integrated NDVI was smaller when simple mechanization was used compared to hand distributed fertilizer, 3) Farms where simple mechanization was used were found to have more stable yields than those who applied inputs by hand.

We found that an increase in the variability of seasonally integrated NDVI of subsamples within farmers’ fields was negatively associated with end of season yield (b_1_ = −10.8, F_1,58_ = 7.7, p < 0.01) (Fig. 1a). If farmers were able to grow a spatially uniform crop that had the yield of their most productive sub-sample, farmers would have achieved yields of 3116 kg ha^−1^. Under the observed heterogeneous stand conditions found in the study, farmers on average yielded 2212 kg ha^−1^. This variability of wheat stands within farmer’s fields caused an average loss of 29% of their potential yield.

The variability of seasonally integrated NDVI was greater when farmers applied inputs by hand when compared with simple mechanization (21 compared to 15), but was not significantly different (p = 0.25) (Fig. 1b). This difference between treatments was more pronounced when comparing end of season yield. Farmers that used simple mechanization had a smaller, more “stable” distribution of yield (p < 0.05). Farms where simple mechanization was used had an inter-quartile range for yield 511 compared to 1293 kg ha^−1^ under hand distributed inputs (Fig. 1c).

### 3.2 Yield response to nitrogen and phosphate rates

**Fig. 2:**
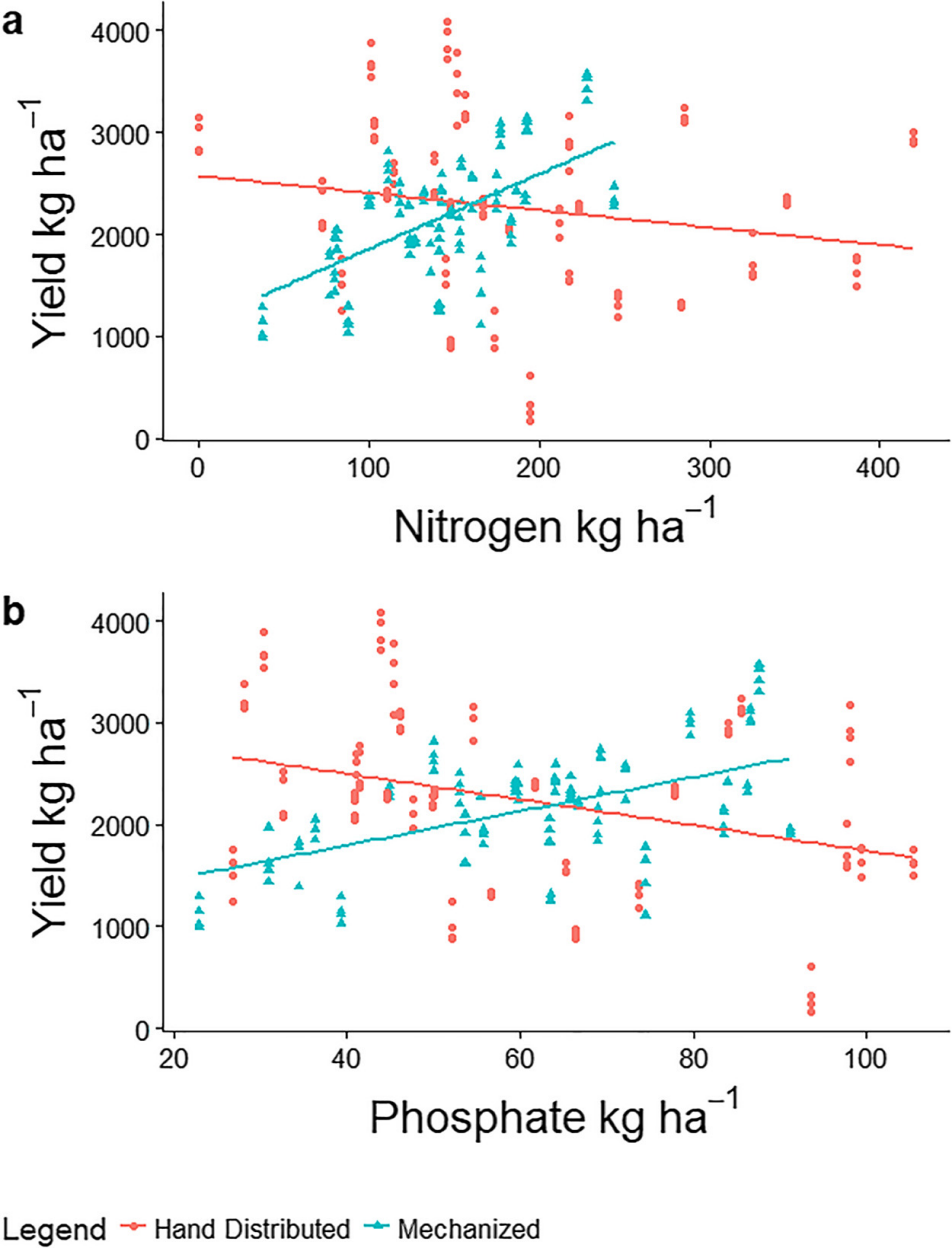
Yield response to fertilizer under two different treatments. a) yield response to N under different treatments indicated that farmers that used simple mechanization had a positive, significant efficiency as opposed to hand distributed fertilizers, b) yield response to P was similar to the response to N in efficiency between the two treatments. Sub-sample estimates of yield per field show variability per field under different treatments.

We found a significant main effect of treatment on the efficiency of N and P fertilizers and end of season yield (p < 0.01 & p < 0.01, respectively). When simple mechanization was used, farmers were able to achieve a significant, positive relationship between their N (b_1_ = 7.3, p < 0.01) and P (b_1_ = 16.7, p < 0.01) fertilizer rates and yield (Fig. 2a & b). In the farmer practice of hand distributed fertilizer application, yield did not respond to increasing N and P rates (b_1_ = −1.6, p = 0.21 & b_1_ = −12.6, p < 0.05, respectively), indicating an inherent inefficiency. Similar relationships between treatment fertilizer efficiency were also observed on seasonally integrated NDVI. There was a strong slope interaction effect between treatments with simple mechanization providing a significant, positive relationship to both N (b_1_ = 0.05, p < 0.05) and P (b_1_ = 0.18, p < 0.01) rates. This relationship was non-significant under hand distributed fertilizer treatment.

### 3.3 Seedling density and yield response to seed rates

A significant main effect indicated there was a difference in the response of seedling density establishment to seed rates under different treatments (p < 0.01). Seedling density increased with an increase in seed rate when simple mechanization was used (b_1_ = 0.74, p < 0.01), while the opposite was true under hand distributed (b_1_ = −0.31, p < 0.05). Additionally, a main effect was observed between treatments for the relationship between yield and seed rate (p = 0.05). Under simple mechanization, yield was found to increase as seed rates increased (b_1_ = 4.4, p = 0.06). As we observed in the relationship between N and P rates and yield under hand distributed inputs, there was a non-significant relationship or negative between seed rates and seedling density establishment and yield (b_1_ = −0.31, p < 0.05 & b_1_ = −1.9, p = 0.21, respectively).

**Fig. 3:**
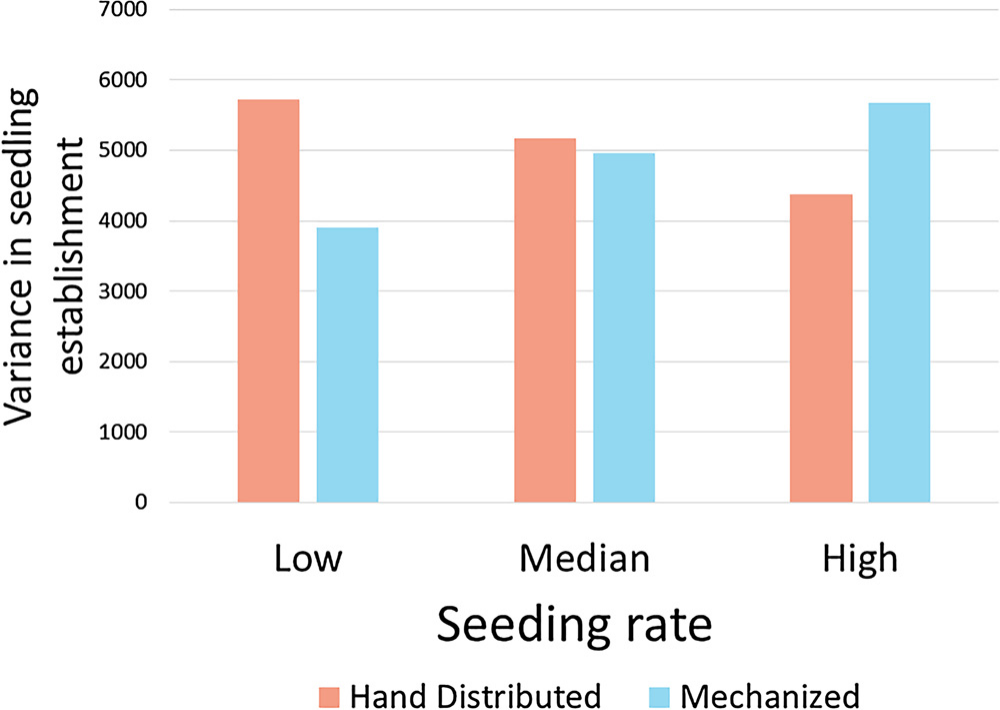
Variation in seedling germination in farmer’s fields at different seeding rates between the two treatments.

The variability of seedling germination was found to be different between treatments when controlling for seed rate (p < 0.05). The response of the variability of seedling germination changed with differing seed rates between the two treatments. At the first quartile and median seed rates, farmers had smaller variability of seedling germination under simple mechanization compared to hand distributed (Fig. 3). At higher seeding rates (third quartile), hand distributed seed had less variability in seedling emergence.

### 3.4 Mechanistic responses to a more precise input application

**Fig. 4:**
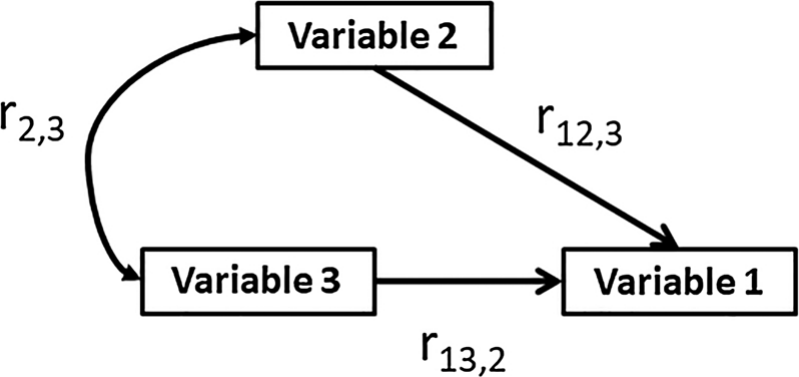
A conceptual framework for models displayed in Table 1.

To better understand why input efficiencies were improved under simple mechanization, we quantified how multiple relationships between agronomic factors changed between treatments. We examined partial correlations of two variables while controlling for a third to better understand the relationships between factors to see if measureable change in their effect on each other was different between treatments. While controlling for the third variable, we determined the correlation coefficient between the controlled variable and the independent variable predicting on the dependent variable. We used yield and seasonally integrated NDVI as our dependent variables. For example, Model 1 in Table 1 describes the relationship of yield (dependent variable) to N rate and seasonally integrated NDVI (independent variables), with estimates shown indicating each independent variable controlling for each other. The covariation between the two independent variables is also provided. A visual representation of these models are shown in Fig. 4. Models 1 through 5 were ordered logically because each subsequent model, i.e. model 2 following model 1 retains at least one independent or covarying factor from the previous model to better explain its results.

**Table 1:** The partial correlations between Variable 1 and Variable 2 controlling for Variable 3 are provided in column r_1,2∼3_. The partial correlations between Variable 1 and Variable 3 controlling for Variable 2 are shown in column r_1,2∼3_. Column r_2,3 (covariance)_ is the correlation coefficient between variables 2 and 3. Explanation of acronyms: Seasonally Integrated Normalized Difference Vegetation Index, SINDVI. †<0.1, *p < 0.05, **p < 0.01, and ***p < 0.001.

Treatment	Model #	Variable 1	Variable 2	Variable 3	r_12,3_	r_13,2_	r_2,3 (covariance)_
Hand distributed	1	Yield	N rate	SINDVI	−0.36†	0.66***	0.08
	2	Yield	Density	SINDVI	0.39†	0.41*	0.6***
	3	SINDVI	N rate	Density	0.18	0.61***	−0.09
	4	SINDVI	Density	Seed rate	0.62***	0.22	−0.31†
Mechanization	1	Yield	N rate	SINDVI	0.33†	0.76***	0.54***
	2	Yield	Density	SINDVI	−0.01	0.74***	−0.03
	3	SINDVI	N rate	Density	0.48**	0.19	0.35†
	4	SINDVI	Density	Seed rate	0.03	0.42*	0.61***

Model 1 establishes the large influence that seasonally integrated NDVI has on yield based on the positive, significant estimates within both treatments. The difference between treatments occurs when we look at the covariance between N rate and seasonally integrated NDVI, whereby they strongly covaried with each other under simple mechanization and do not under hand distributed.

Model 1 indicated that seasonally integrated NDVI strongly predicts end of season yield shown by positive, significant estimates for both mechanized and hand distributed treatments (b1 = 0.76, p < 0.001 & b1 = 0.066, p < 0.001, respectively). However, under mechanization N rate and seasonally integrated NDVI also covaried (b1 = 0.54, p < 0.001) whereas this was not the case when hand distribution was used (b1 = 0.08, p > 0.1). Model 2 provided evidence that plant density effectively traded roles with N rate from model 1 between treatments, and that plant density strongly covaried with seasonally integrated NDVI under hand distributed rather than the mechanized treatment (b1 = 0. 6, p < 0.001 & b1 = 0.-03, p > 0.001, respectively). Model 3 provided validation of the results from models 1 and 2, indicating that N rate was a strong predictor of seasonally integrated NDVI under simple mechanization (b1 = 0.61, p < 0.001), and conversely that plant density fulfilled this role under hand distributed (b1 = 0.48, p < 0.05). Model 4 indicated that when hand distributed, seed rate became disconnected from seedling density by the lack of a significant covariance (-0.31, p > 0.10). Seedling density and seed rate covaried when simple mechanization is used (0.61, p < 0.001).

### 3.5 Labor efficiency

Simple mechanization was 52% faster at applying the same rate of seed compared to applying it by hand (F_1,38_ = 76.8, p < 0.01). Farmers that distributed fertilizer by hand took an average of 2.1 h to complete sowing a hectare, while this value was 1 h using simple mechanization.

### 3.6 Profit stability and return on investments

**Fig. 5:**
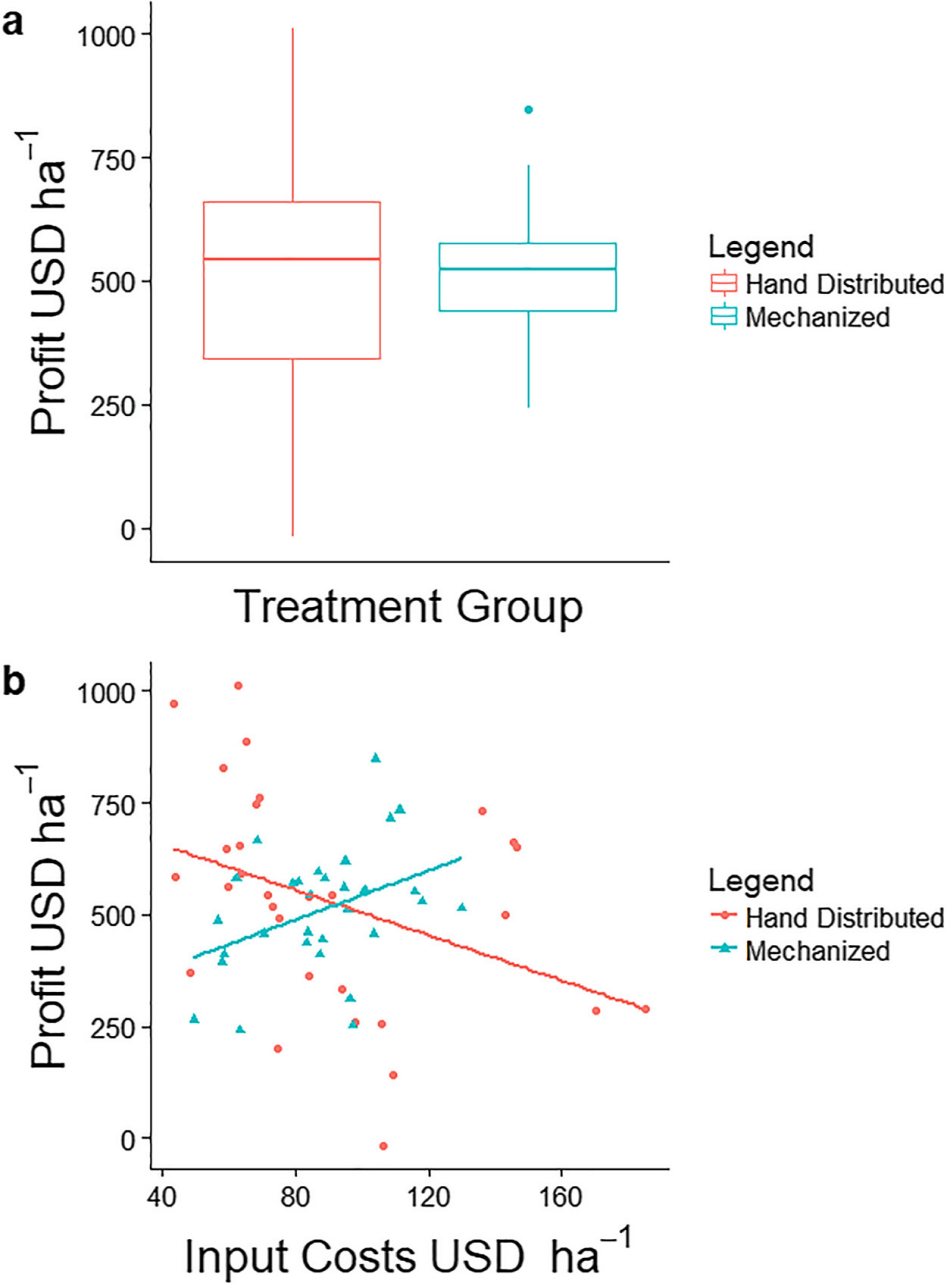
a) distribution of profits between two the two treatments indicate that simple mechanization provided more predictable profits, b) response of profit to increasing input costs was positive with simple mechanization, but negative when inputs were distributed by hand.

Farmers that used simple mechanization had more predictable profits than those applying inputs by hand as indicated by a smaller inter-quartile range of $140 USD compared to $318 (p < 0.05) (Fig. 5a). Even though farmers that applied inputs by hand used more fertilizer compared to simple mechanization, profits were equivalent between the treatments ($530 compared to $509) (F_1,56_ = 0.17, p = 0.68). Similar to the relationship between N, P and yield, we found that there was a significant interaction effect among treatments between fertilizer rates and farmer profits (F_1,56_ = 8.2, p < 0.01 & F_1,56_ = 13.1, p < 0.01, respectively). We found that when simple mechanization was used, profits increased with rate increases of N (b_1_ = 2.8, F_1,28_ = 9.1, p < 0.01) and P (b_1_ = 3.7, F_1,28_ = 8.3, p < 0.01). When fertilizer was applied by hand, increasing N rates (b_1_ = −1.7, F_1,28_ = 3.9, p = 0.05) or P (b_1_ = −0.9, F_1,28_ = 3.5, p = 0.06) did not increase partial-profit. There was no significant interaction effect among seed rates and profits between the treatments (F_1,56_ = 3.5, p = 0.07).

With more predictable profits, and a greater return on investment from fertilizer with simple mechanization, we found that there was a significant difference among treatment groups between cost of inputs (N, P, and seed) and profits (F_1,56_ = 7.2, p < 0.01). Farmers in the simple mechanization treatment group had a positive relationship between the costs associated with inputs and profit (b_1_ = 2.8, F_1,28_ = 5.2, p < 0.05), while the opposite was true for the treatment group using hand distributed inputs (b_1_ = −2.5, F_1,28_ = 4.9, p < 0.05) (Fig. 5b).

### 3.7 Growing conditions section

Yield was affected by different environmental conditions found across the 60 farms in the study, but the influence of these factors on yield were not found to have significant interaction effects between treatment groups. Soil texture classifications ranged between clayey to silty loam soils, with both the percentages of clay and silt found to affect yield (F_1,56_ = 5, p > 0.05 & F_1,56_ = 3.8, p = 0.055, respectively) while indicating no interaction between the mechanization and hand distributed treatments (F_1,56_ = 0.2, p = 0.65 & F_1,56_ = 0.35, p = 0.57, respectively). The effect of seasonal abstraction of water through the soil profile did not have an effect on yield, nor was there an interaction effect among treatments (F_1,56_ = 0.165, p = 0.67 & F_1,56_ = 0.46, p = 0.5, respectively). The slope of the Crop Water Stress Index was found to not impact end of season yield, nor was there a treatment interaction effect (F_1,56_ = 3.5, p = 0.07 & F_1,56_ = 0.72, p = 0.4, respectively). Atmospherictemperatures above 31 °C constituting terminal heat stress (Al-Khatib and Paulsen, 1984) did not occur around the time of anthesis within our study.

## 4 Discussion

### 4.1 The benefits of precision, cost of imprecision

The use of simple mechanization provided farmers with multiple advantages over the traditional practice of applying inputs by hand. Foremost of these benefits was the reduction of variability of yield, offering farmers more predictable, stable yields. Although the difference between treatments with respect to the variability of seasonally integrated NDVI was not statistically significant, the significant improvement in yield stability and improved relationships within covariance model 1 demonstrated that the difference between treatments was biologically significant. As we saw in the response of yield to N and P rates under the two treatments, using simple mechanization led to a more homogenous spatial distribution of inputs across the field and therefore increased the likelihood that each wheat plant had access to these fertilizers during the growing season. Conversely, our data suggests that applying fertilizer by hand introduced a measure of spatial unpredictability and local aggregation in fertilizer distribution throughout farmer fields. Uneven distribution of fertilizer across the field led to unequal nutrient availability to individual plants, thereby limiting healthy growth, and ultimately yield.

Our results indicated that the higher yield stability found under simple mechanization was a response to increasing the strength of the relationship between fertilizer and yield. Fig. 2a & b shows the positive, predictable yield response for fertilizer rates when they are applied with added precision. This was also observed in model 1 where there was a strong relationship between seasonally integrated NDVI and yield that was mediated by N rate when simple mechanization was used. This observed relationship between yield and seasonally integrated NDVI mediated by N rate is what would be expected in a farming system with uniform plant access to fertilizer (Labus et al., 2002; Teal et al., 2006). The relationship between fertilizer, yield and seasonally integrated NDVI was weakened when inputs were applied by hand, with model 2 indicating that seedling density became the main predictor of seasonally integrated NDVI rather than N rate when inputs were hand distributed.

The disassociation of yield to increasing rates of N in the hand distributed treatment was particularly surprising because this group of farmers applied 18% more N (F_1,56_ = 3.6, p = 0.06) than farmers using simple mechanization. This challenged our expectation that the impacts to yield from a non-uniform application of fertilizer could be overcome by increasing fertilizer rates, and points to the decrease in efficiency that occurs when inputs are traditionally applied. Additionally, lower intercepts for both N and P on farms using simple mechanization (Fig. 2a & b) provided evidence that this treatment group may have been on poorer quality land, and that simple mechanization may have helped overcome even this limiting factor of the production system.

A more consistent response to fertilizer was achieved using simple mechanization even though farmers that distributed inputs by hand used greater rates for two of the three macronutrients. This includes not only more N, but 11 of the 30 farmers in the group applied an average of 24 kg ha^−1^ of K while farmers in simple mechanization treatment applied none. Potassium has been found in multiple studies to be an underused, but critical farm input towards improved wheat productivity in South Asia (Ladha et al., 2003; Park et al., 2018). Even with more inputs used in the hand distributed treatment, there was no statistical difference in the magnitude of yield between simple mechanization and hand distributed treatments (2168 compared to 2255 kg ha^−1^) (F_1,56_ = 0.22, p = 0.64).

The covariance models provide added context for efficiency gains we saw in Fig. 2a (these relationships held with P rates as well). Because NDVI is strongly associated with fertilizer uptake and crop vigor (Hansen and Schjoerring, 2003), the absence of a significant influence of N rate on seasonally integrated NDVI under hand distributed treatment was troubling. This suggested that when fertilizer is applied by hand, the relationship between N rate and seasonally integrated NDVI became weak enough that stand density became the de facto agronomic factor which best predicted seasonally integrated NDVI. These results underscored the reality that farmers using traditional hand distributed methods were forfeiting yield to inefficiency caused by non-uniform application of fertilizers. In covariance models 1 and 3, simple mechanization showed evidence of improving the relationship of fertilizer to yield and seasonally integrated NDVI.

The benefits observed from using simple mechanization with respect to seedling density establishment, reduction of seedling density variability under low and median seed rates, and improving the relationship between seed rate and yield paralleled those found with fertilizer efficiencies. Similar benefits of precision agriculture have been observed in the rice-wheat cropping systems of the Indian states of Uttar Pradesh and Bihar adjacent to the study site, where zero-till has increasingly replaced hand distributed practice (Erenstein and Laxmi, 2008; Keil et al., 2015). Seed rates in our study were high relative to averages in Bihar, India which shared similar environments and cropping system (Pathak et al., 2003), with farmers across both treatments in our study applying 37% more seed per hectare compared to farmers in Bihar (184 compared to 116 kg ha^−1^) (Park et al., 2018). The higher rates of seed used in Nepal may reflect a risk reduction strategy by farmers to try and overcome the poor relationship we observed between seed rates, seedling establishment, and yield when applied by hand.

Unfortunately, the apparent assumption made by farmers that adding more seed leads to greater seedling establishment and yield does not appear to be a good risk management strategy when not paired with precise sowing. This is because, as we observed in covariance model 4, seedling density and seed rate covaried when simple mechanization was used, which was not the case when seed was hand distributed. Higher adoption of zero-tillage in Bihar may be partially responsible for lower seed rates, in part because direct seeding adds a measure of precision to sowing thereby reducing the need for more seed, but rather relying on mechanization to improve the efficacy of a lesser rate (Erenstein and Laxmi, 2008; Keil et al., 2015).

We believe the simple mechanization we implemented in our study could provide a pragmatic, lower-cost intermediate practice between the low precision conventional practice of hand distributed seed and fertilizer paired with incorporation by cultivation and/or rotovation, and the higher precision direct seeding and fertilizing of zero tillage. This widespread use of zero tillage in Bihar, and across other Indian states was a decades long product of both an active government and non-governmental organizations presence in the agricultural sector, and also the development a strong private network of service providers that offer zero tillage service (Keil et al., 2015, 2016). As with fertilizer and labor availability, it is therefore unlikely a change from rotovation and cultivation to zero tillage, and the precision it brings to the farmer’s production system, will occur in the near future in Nepal. Simple mechanization offered multiple production advantages over traditional practices through improved input efficiencies, all the while fitting within the traditional semi-mechanized system necessary towards scaling up potential adoption (George, 2014).

### 4.2 Assessing unexplained variation in study

Within hand distributed treatment the negative response of density to seed rate, and a changing response in the changing variability of seedling germination under different seed rates, and the negative or non-significant responses of N and P to yield was puzzling. Some farmers applying inputs by hand had excellent yields, while others had poor yields. While our hypothesis was that a more uniform application of inputs would reduce intra-field variability, we suspected that the poor efficiencies we observed under hand distributed inputs were interacting with unknown sources of variability associated with environmental heterogeneity within a farmer’s field. The clearest evidence of this source of stress are the varying levels of germination variability with different seed rates (Fig. 3), and covariance model 4. We suspect that farmers applying seed by hand at the third quartile seed rate achieved a lower variability of seedling germination in part because they were applying variable seed rates to areas prone to higher seedling stress, thereby helping to offset expected seedling die off with higher seed rates. The absence of covariation between seed rate and seedling density in covariance model 4 within the hand distributed treatment is perhaps indicative of this environmental variability as well. This source of variation when combined with the lack of uniformity of seed application may have led to higher die off of seedlings because of the higher risk of aggregation into parts of field with higher risk of stress during germination. Conversely, it appeared that precision application of inputs minimized the influence of this unexplained variability to the point at which positive efficiencies could be achieved. This can be observed in the positive relationship between seedling establishment and seed rate when simple mechanization was used under similar growing conditions as the hand distributed treatment.

The source or sources of variation that mitigated the relationships between yield, seasonally integrated NDVI, and N and the other relationships in Table 1 were most likely associated with soil-water-plant interactions. Differences in fertilizer availability to plants has been attributed to the pH of the soil (Sanyal and Datta, 1991) and variable fertilizer sorption rates due to differences in the drying of soil profiles (Sah and Mikkelsen, 1986). Waterlogging stress is associated with poorly drained soils can also prevent uptake nutrients and water in wheat (Luxmoore and Stolzy, 1969) which can reduce end of season yield (Trought and Drew, 1980). Landscape position can influence these relationships as well (McDonald et al., 2006). Stresses within soil-water-plant interactions may also help explain the disassociation between seed rate and seedling density (Grable, 1966). Further investigation into this source of variability would provide better context as to why precision agriculture appears to alleviate it, as well as identify practices to solve it directly.

### 4.3 Addressing a declining labor pool with higher labor efficiency

Our results demonstrated that simple mechanization was a labor saving technology that doubled the labor efficiency of the input of application process when compared to traditional hand distributed practices. An improvement in labor efficiency could help alleviate bottleneck and labor problems of a shrinking agricultural workforce and associated knowledge base that is not being replaced with supplemental labor or mechanization in an environment that penalizes yield if key farming operations are delayed. This can also facilitate more timely farm operations during labor bottlenecks such as early sowing and top dressing of N.

The mechanized treatment also demonstrated that the agricultural knowledge and skillsets of laborers can also be effectively mechanized. Farmers that distributed inputs by hand had on average 22 years of experience honing this skill. Prior to implementation of this study, our researchers had no previous experience using the simple mechanization tool in order to best simulate a new user. The gains in seed, fertilizer and labor efficiency we observed under the simple mechanization treatment with no previous experience demonstrates that precision agriculture via mechanization effectively replaced skilled agricultural labor during the input application stage of farming operations.

### 4.4 Increasing the predictability of profit for smallholders

Introducing simple mechanization provided farmers not only with more predictable profit, but also greater stability of return on investment of their inputs. We believe that from a farmer’s perspective, these results can be interpreted to represent less risk to their bottom line when considering the scarcity of access to inputs (Gollin, 2006). Farmers that hand applied their inputs faced higher risk in their wheat production, as we saw no evidence that adding more inputs benefited their end of season yield and profit. Our results highlight that low fertilizer application rates in Nepal may not only be a byproduct of poor government policy and limited private sector development, but likely hesitation on farmer’s part because they historically have seen little profit response to higher rates of inputs under the widely adopted hand distributed technique. This could also help explain why farmers do not reinvest remittance dollars into more farming inputs (Maharjan et al., 2013). Introducing technologies like the spreader evaluated in this study that improves the relationship between inputs and profit can increase farmer confidence in investing in their farms, which in the long-term could help improve productivity and profitability of Nepali agriculture.

## 5 Conclusion

Adding precision to fertility and seed placement by simple mechanization was an improvement over traditional practices in four distinct ways: 1) increased input efficiency with respect to yield and seedling density, 2) doubled labor efficiency during the input application process, 3) reduced variability of yield within and between fields, and 4) assured farmers returns on investment from their inputs. Our analysis indicated that many of the variability and human error problems associated with traditional hand distributed practices can be overcome with the addition of relatively simple mechanization, while still fitting within semi-mechanized tillage system. Solutions like the chest-mounted spreader can offer a low-cost precision agriculture stopgap between traditional practices and larger mechanization like zero tillage while still offering more timely farming operations and better labor and input efficiencies. Increasing the return of investment to farmers may help reverse the chronic underinvestment in farm operations observed in Nepal. Countries like Bangladesh, Sri Lanka, Thailand, Vietnam, and Uganda still use hand distribution of farm inputs small grains cropping systems and likely suffer inefficiencies as a result. Simple mechanical solutions like our intervention may provide similar benefits in these countries, and we recommend that they be tested in production environments with similar labor and efficiency problems.

## Funding

This work was supported by the Bill & Melinda Gates Foundation, Seattle, WA [grant number OPP1052535]; the United States Agency for International Development, Washington D.C. [grant number BFS-G-11-00002]; and the United States Borlaug Fellows in Global Food Security Awarded to Purdue University, Washington D.C. [grant number A11023.2].
